# Machine vision-based detection of key traits in shiitake mushroom caps

**DOI:** 10.3389/fpls.2025.1495305

**Published:** 2025-02-03

**Authors:** Jiuxiao Zhao, Wengang Zheng, Yibo Wei, Qian Zhao, Jing Dong, Xin Zhang, Mingfei Wang

**Affiliations:** ^1^ Intelligent Equipment Technology Research Center, Beijing Academy of Agriculture and Forestry Sciences, Beijing, China; ^2^ Information Technology Research Center, Beijing Academy of Agriculture and Forestry Sciences, Beijing, China

**Keywords:** shiitake mushroom breeding, edge detection, machine learning, OpenCV model, phenotypic key features

## Abstract

This study puts forward a machine vision-based prediction method to solve the problem regarding the measurement of traits in shiitake mushroom caps during the shiitake mushroom breeding process. It enables precise phenotyping through accurate image acquisition and analysis. In practical applications, this method improves the breeding process by rapidly and non-invasively assessing key traits such as the size and color of shiitake mushroom caps, which helps in efficiently screening strains and reducing human errors. Firstly, an edge detection model was established. This model is called KL-Dexined. It achieved an per-image best threshold (OIS) rate of 93.5%. Also, it reached an Optimal Dynamic Stabilization (ODS) rate of 96.3%. Moreover, its Average Precision (AP) was 97.1%. Secondly, the edge information detected by KL-Dexined was mapped onto the original image of shiitake mushroom caps, and using the OpenCV model,11 phenotypic key features including shiitake mushroom caps area, perimeter, and external rectangular length were obtained. Experimental results demonstrated that the R² between predicted values and true values was 0.97 with an RMSE as low as 0.049. After conducting correlation analysis between phenotypic features and shiitake mushroom caps weight, four most correlated phenotypic features were identified: Area, Perimeter, External rectangular width, and Long axis; they were divided into four groups based on their correlation rankings. Finally,M3 group using GWO_SVM algorithm achieved optimal performance among six mainstream machine learning models tested with an R²value of 0.97 and RMSE only at 0.038 when comparing predicted values with true values. Hence, this study provided guidance for predicting key traits in shiitake mushroom caps.

## 1 Introduction

Shiitake mushroom have antimicrobial, antitumor, antiviral, anti-inflammatory and antioxidant properties (Ahmad et al., 2023). Global shiitake production accounts for nearly 22% of the total mushroom production (Kumar et al., 2022). China is the world’s largest producer, consumer and exporter of edible mushrooms (Li and Xu, 2022), in which the industrial production of shiitake mushrooms has shown a rapid growth, and the production of shiitake mushrooms in China will be about 12.9 million tons in 2021, which is more than 90%of the world’s total production (Wang et al., 2024). Shiitake breeding is the most important link in the whole shiitake industry chain, and the yield and quality of shiitake can be improved by selecting shiitake varieties with excellent genetic characteristics. Measuring key traits of shiitake mushroom caps is an important step in shiitake breeding, which helps to evaluate and select shiitake varieties with desirable characteristics (Zhang et al., 2022). And among them, shiitake mushroom caps weight trait is not an isolated trait characteristic, it is closely related to other key phenotypic traits such as shape, color, and texture. Therefore, it is very meaningful to carry out machine vision-based prediction of key traits in shiitake mushroom caps.

The prerequisite for accurately detecting key indicators of shiitake mushroom caps phenotypes is the accurate positioning of edges. Some edge detection methods based on traditional approaches have been found. Zhang et al. (2021) proposed an improved Canny algorithm, which utilizes the Sobel operator and approximation method to calculate gradient size and direction, and introduces an adaptive thresholding algorithm to determine the image threshold. This algorithm can effectively detect image edges without requiring threshold adjustment, with a detection time of only 1.231 ms. Chen et al. (2024) proposed an effective detector for small-size defects on fabric surfaces, which utilizes a trunk with attention mechanism to enhance the acquisition of location information regarding small-size defects. The FPN+PAN multiscale detection structure is used to effectively integrate feature information across different levels. The results show that the detection recall and accuracy in the public fabric dataset reach 0.56 and 0.842 respectively, while reducing false detection rate of small-size defects by 2.7% compared to other detectors. Gao et al. (2010) proposed a method that combines the Sobel edge detection operator with wavelet denoising to perform edge detection on images containing Gaussian noise. Compared to the traditional method based on a single detection operator, its effect on edge detection is more pronounced. In recent years, with the development of deep learning technology, edge detection methods based on deep learning have gradually become mainstream. Su et al. (2021) proposed PiDiNet, a simple and lightweight yet effective architecture for efficient edge detection. Extensive experiments were conducted on BSDS500, NYUD, and Multicue datasets, and the results demonstrated its efficient inference efficiency. Jie et al. (2024) introduced EdgeNAT, a Transformer-based single-stage edge detector with DiNAT as an encoder that can accurately and efficiently extract object boundaries and edge features. The algorithm exhibits good performance in both single-scale input and multi-scale input scenarios. Lin et al. (2023) designed MI-Net, a multi-level interactive contour detection model that combines convolutional neural network and self-attention mechanism by drawing inspiration from the effective mechanisms of biological vision systems for contour detection. Experiments conducted on public datasets demonstrate excellent performance of this method. However, neither traditional nor deep learning-based edge detection methods have been widely applied in shiitake mushroom caps analysis due to challenges posed by unstructured patterns on the surface of shiitake mushroom caps as well as background variations.

Regarding the prediction of shiitake mushroom caps weight, there are fewer research applications in this area at this stage, and some methods for weight prediction exist in other fields. He et al. (2023) proposed LiteHRNet (Lightweight High-Resolution Network), which applied their method using RGB-D images to estimate the live weight of sheep, resulting in a Mean Percentage Error (MAPE) of 14.605%. Wang et al. (2023) developed a machine vision-based technique to extract feature information from images of crab abdomen and back by pre-processing the images and combining them with genetic algorithm and back-propagation algorithm to construct a crab quality grading model with an accuracy of up to 92.7%. Mirbod et al. (2023) utilized stereoscopic cameras to convert segmented apple shapes into diameters, achieving mean absolute errors ranging from 1.1 to 4.2 mm, with a mean error of 4.8%compared to ground truth diameter measurements. He et al. (2023) proposed an improved YOLOv5 model that utilizes a coordinate attention module and a bounding box regression loss function to detect and accurately count pod targets on soybean plants. The mean squared error (MSE) for the estimation of the weight of a single pod was 0.00865, and the mean relative error for total weight estimation of all potted soybean plants was 0.122. In summary, 2D weight estimation methods are not effective in estimating the weight of shiitake mushroom caps due to the limited number of input terms, while 3D imaging-based weight estimation is not suitable for shiitake mushroom caps weight estimation due to the high cost of equipment and deployment difficulties.

In summary, this study proposes the use of machine vision to analyze the phenotypic information of shiitake mushroom caps and predict their weight based on key phenotypic data, aiming to achieve high-throughput and high-precision prediction of important traits. The main objectives of this study include three aspect.

(1)Establishing a lightweight machine vision algorithm to efficiently capture the edge features of shiitake mushroom caps and evaluate its performance under red and green backgrounds;
(2)Identifying the four most relevant phenotypic key features based on their correlation with the weight of shiitake mushroom caps;
(3)Developing default input terms for efficient estimation of shiitake mushroom caps weight and accurately estimating it in real-time using a machine learning model specifically designed for shiitake mushrooms.

In “Section 2 Materials and Methods”, this study will focus on the construction of the experimental setup, the acquisition of data, and the composition of the algorithms. In “Section 3 Results”, this study will verify the feasibility of our approach through experiments, as well as validate the performance of the image algorithms and machine learning algorithms. In “Section 4 Discussion”, this study discuss and analyze the significant experimental findings from the trial. In “Section 5 Conclusions”, this study summarize the conclusions drawn from our experiments.

## 2 Materials and methods

### 2.1 Testing apparatus

**Figure 1:**
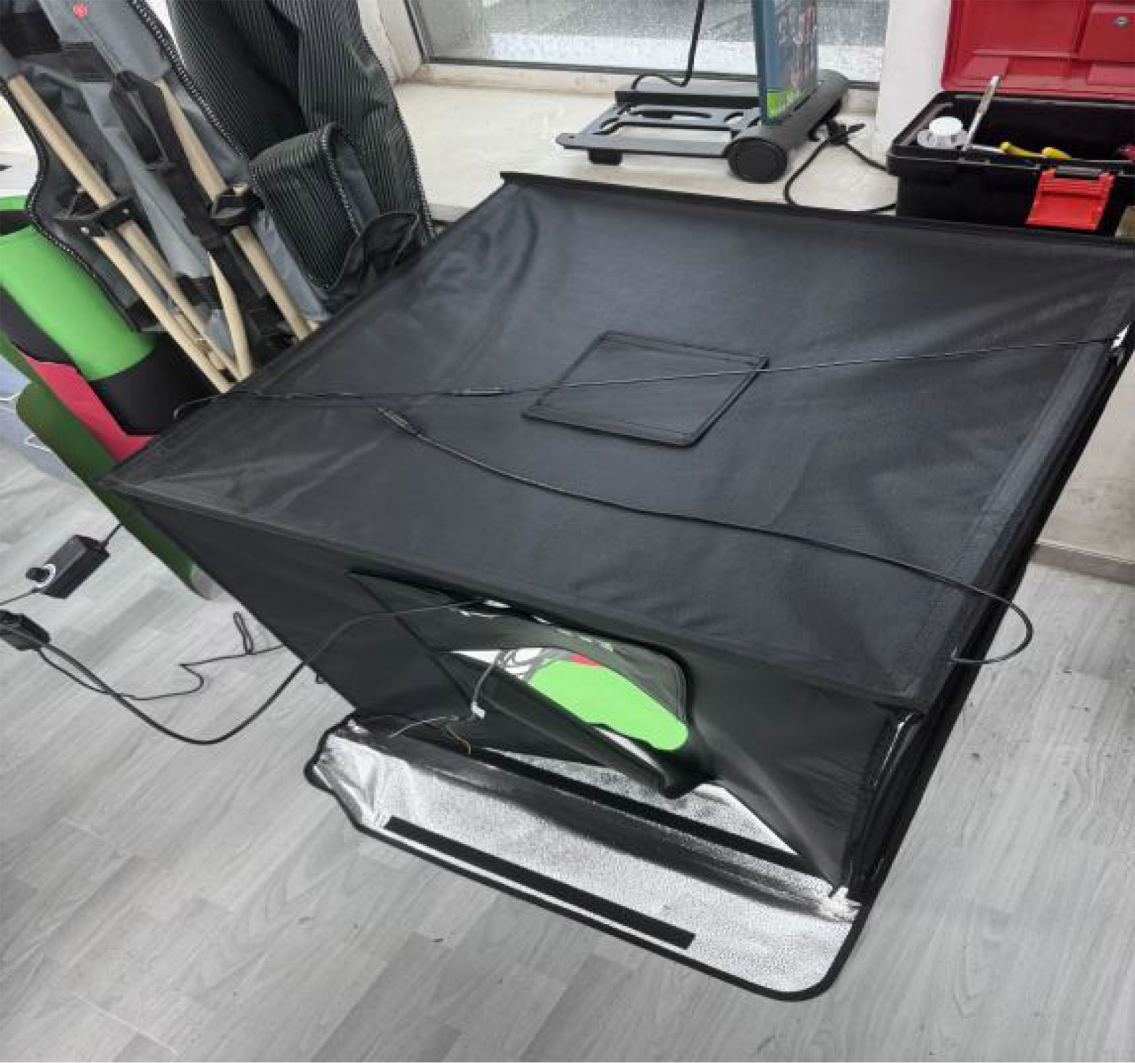
Photograph of the device.

**Figure 2:**
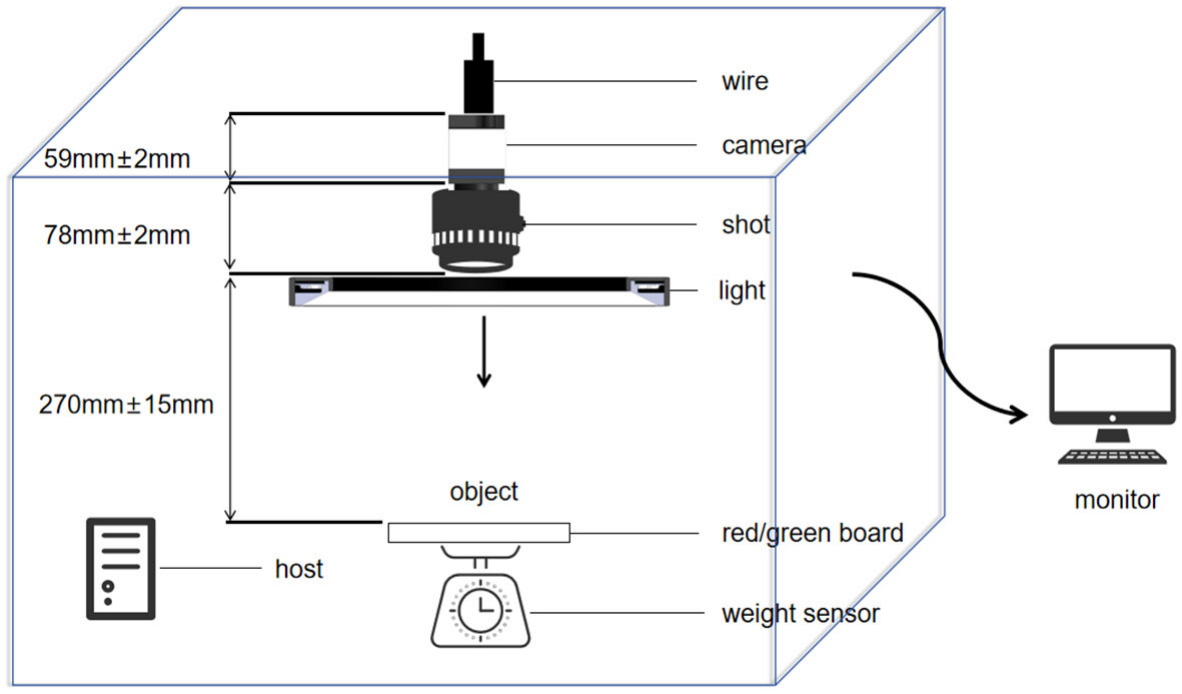
Schematic diagram of the interior of the phenotype resolution device.

The experimental site was located at the National Precision Agriculture Demonstration Base in Changping District, Beijing, China, at the coordinates of 116.46°E longitude and 40.18°N latitude. The data samples were provided by the Institute of Plant Protection of Beijing Academy of Agricultural and Forestry Sciences as well as the Institute of Edible Mushrooms of Shanghai Academy of Agricultural Sciences, and the sample variety was Jingxiang 118. The exterior of the device is shown in **Figure 1**, and the internal structure is shown in **Figure 2**. The image acquisition device consists of an interface (camera and host interface selected USB interface, model:USB3-1-4-2-5M-Z), box (DEEP/80CM×80CM×80CM), camera (model MV-S2UC2000GM with a resolution of 20 megapixels),lens(fixed-focus lens MV-LD-16-20M-B with a medium image element for high resolution global exposure), background plate (red/green), fill light (120W four-color light source lamp with a color temperature of 5500K),USB expansion port, host computer (11th Gen Intel (R) Core (TM) i5-11400F),monitor (LG), pressure sensor (Model:8101-300g with an accuracy levelof0.1g). The internal schematic diagram of the phenotype resolution device is shown in **Figure 2**. In this study, the objective distance between the lens and the subject was set to be 300mm, and the field of view was 230mm×150mm. This configuration allows for clearer acquisition of shiitake mushroom caps images.

### 2.2 Data

#### 2.2.1 Image data

**Figure 3:**
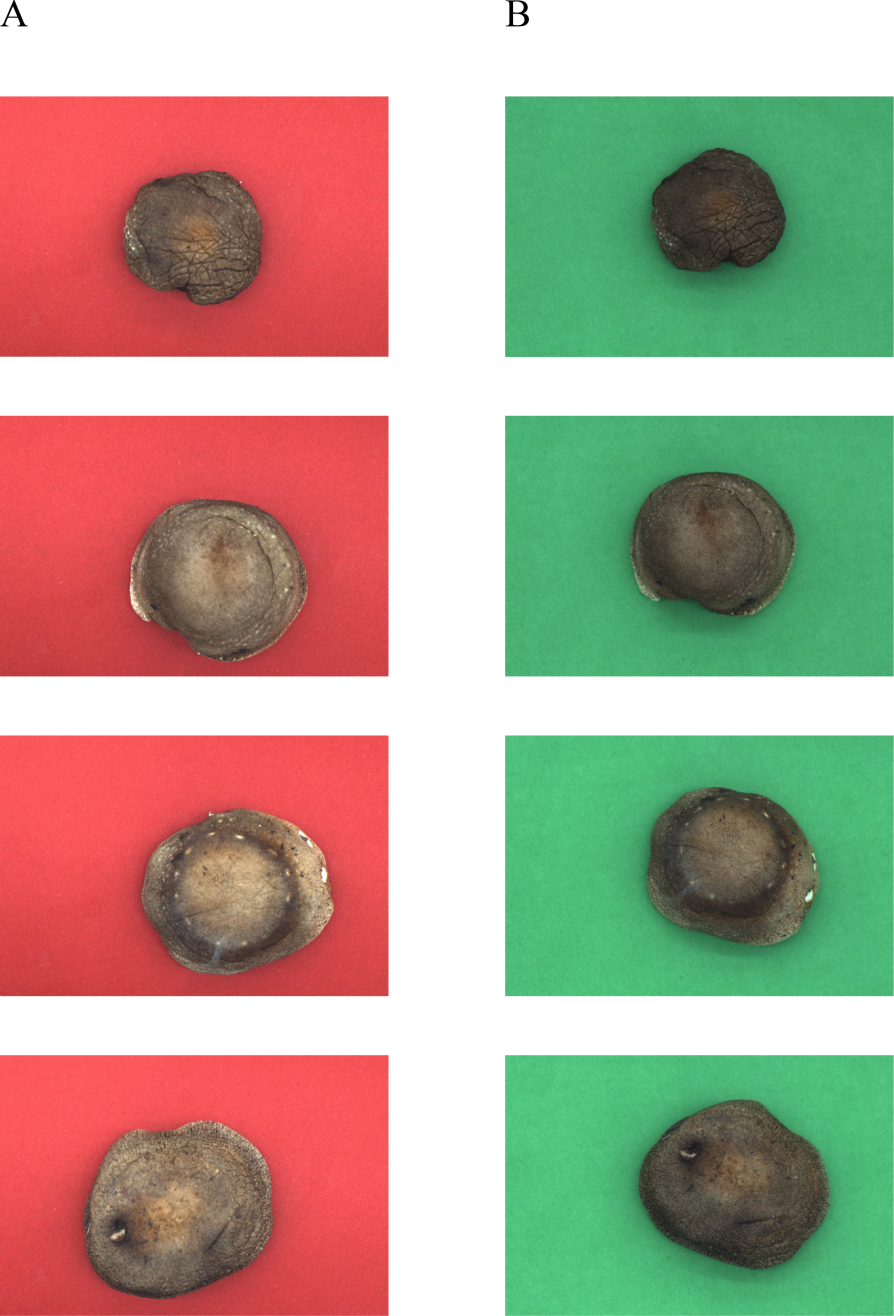
Photographs of shiitake mushrooms in different background conditions. **(A)** Red-shiitake mushroom caps. **(B)** Green-shiitake mushroom caps.

The box (DEEP/80CM×80CM×80CM) plays a crucial role in controlling image quality. It utilizes a surface light source to ensure the uniformity of illumination, which is essential for creating consistent lighting conditions across the object being photographed and avoiding potential distortions in the captured images. Additionally, the interior of the box is fully covered with reflective materials. This serves to reduce the interference caused by the light source as the reflected light is redirected in a more controlled way, minimizing unwanted reflections or glare that could otherwise degrade the image quality. Moreover, within this stable and controlled environment provided by the box, the exposure of the industrial camera can be adjusted. By setting the appropriate exposure, the camera is able to capture images with the right brightness, contrast, and clarity. Altogether, these measures within the box are vital for obtaining high-quality images that are necessary for accurate subsequent image analysis and the extraction of precise information related to the objects under examination. The photographs in **Figure 3** depict shiitake mushroom caps captured under various background conditions between June 2024 and August 2024. The study utilized 686 shiitake mushroom caps from 10 batches of the shiitake variety Jingxiang 118. These samples were placed inside a camera box, with a designated region of interest (ROI) for image acquisition. Subsequently, the shiitake mushroom caps were photographed using an industrial camera’s snapshot method, employing a perpendicular angle of incidence and an image resolution of 2736 x 1836 pixels. Ultimately, a collection of 686 pictures was obtained for both red and green backgrounds, resulting in two datasets: Green-shiitake mushroom caps and Red-shiitake mushroom caps.

#### 2.2.2 Key traits of shiitake mushrooms caps

Based on the measurement outline of shiitake mushrooms from the Institute of Edible Mushrooms of the Shanghai Academy of Agricultural Sciences, the key trait characteristics of the collected shiitake mushroom cap samples were measured and analyzed in detail in this study (Deng et al., 2024). **Table 1** lists the key trait indicators, measurement methods, units and measurement standards used in this study. These key trait indicators not only included the physical dimensions of shiitake mushrooms caps, but also covered the indicators of R, G and B channel colors and weights, in order to comprehensively assess various key trait characteristics of shiitake mushrooms caps. The external rectangular length and width of shiitake mushrooms caps were measured using vernier calipers in millimeters (mm).To ensure the accuracy and reproducibility of the data, three independent measurements of the length and width of each shiitake mushrooms cap were taken and averaged as the final result (Wang et al., 2018). Measurements of the circumference and long axis of shiitake mushrooms caps were also taken by manual vernier caliper method and followed the same method of averaging three measurements to ensure the accuracy of the data. The table also mentions the color parameters of the shiitake mushrooms caps including the mean values of the red (R), green (G) and blue (B) color channels as well as the grey scale mean values. The color parameters were extracted from the photographs of shiitake mushrooms caps by using image analysis software (Mindvision SDK for windows V2.1.10.185) and they reflect the characteristics of color distribution on the surface of shiitake mushrooms caps. The mean values of the color parameters help to understand the possible color variations of shiitake mushrooms caps under different cultivation conditions, which are eventually compared with them using standard color cards. Finally, the weight of shiitake mushrooms caps is an important index in this study, which can reflect the yield and growth of shiitake mushrooms caps, and a standard weight of 50g-200g was chosen to validate the values measured by the weight sensor (Seligman et al., 2023), as all the weights of shiitake mushrooms caps used in this study were within this range.

**Table 1:** Key traits of shiitake mushrooms caps.

Key traits of shiitake mushrooms caps	Measurement mode	Unit	Measured standard
External rectangular length	Dial calipers	mm	Average of 3 measurements
External rectangular width	Dial calipers	mm	Average of 3 measurements
Roundness	—	—	Ratio of length to width
Area	—	—	—
Perimeter	—	—	—
Long axis	Dial calipers	mm	Average of 3 measurements
Short axis	Dial calipers	mm	Average of 3 measurements
Red mean	Standard Color Cards	—	—
Green mean	Standard Color Cards	—	—
Blue mean	Standard Color Cards	—	—
Greyscale mean	—	—	—
Weight	Weight sensors	g	50-200g weights

### 2.3 Method

**Figure 4:**
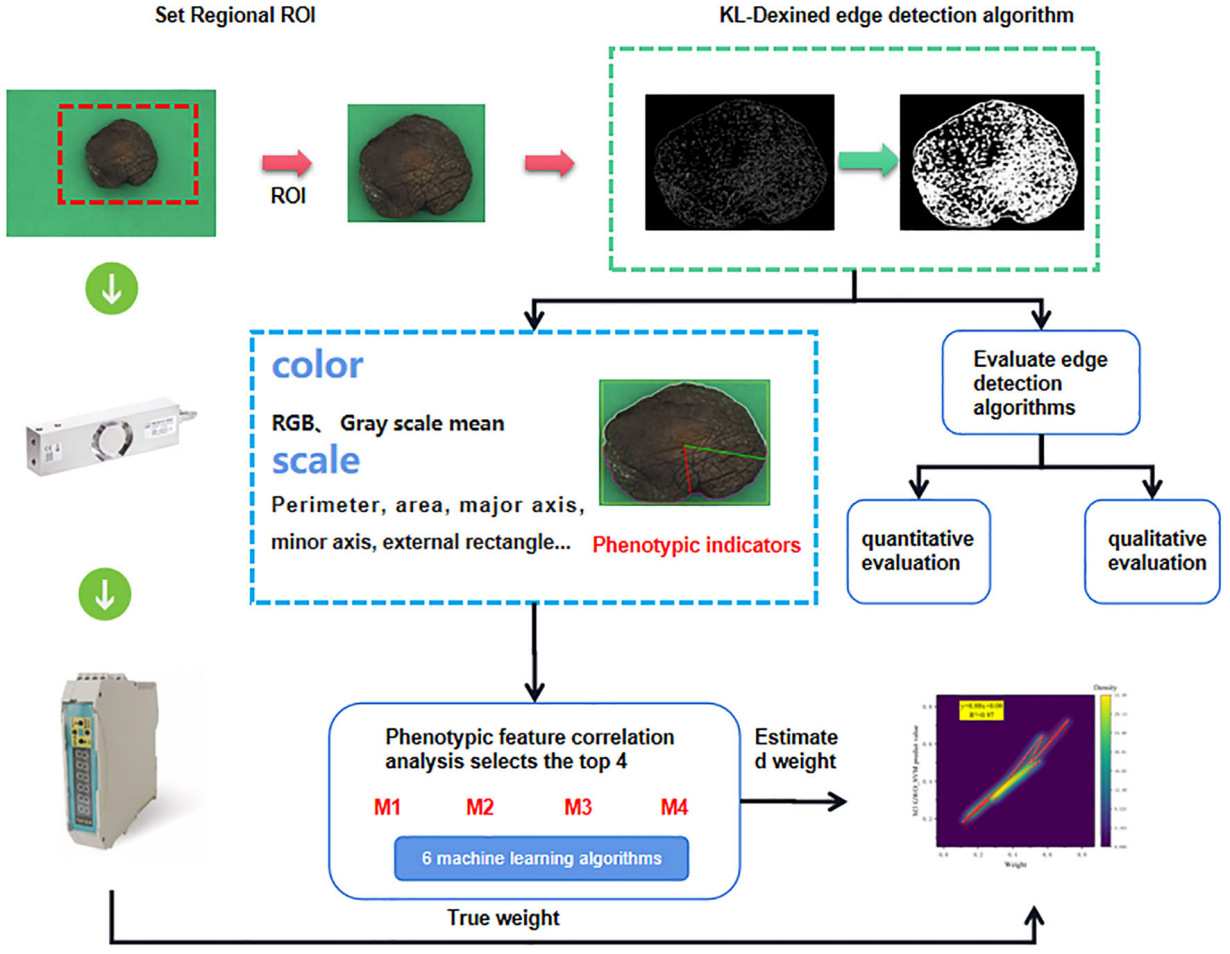
General structure diagram.

In this study, the accurate edge detection algorithm is used to resolve the edge of shiitake mushroom caps and obtain their phenotypic information through the KL-DexiNed edge detection network. Simultaneously, a connection between weight and phenotypic information is established to compare the performance of different machine learning algorithms with various input group items. The model framework diagram is shown in **Figure 4**. Specifically, (1) pictures of shiitake mushroom caps are collected using phenotypic acquisition equipment, and an improved deep learning-based edge detection algorithm is employed to determine the scale indicators of their phenotypes; (2) weight estimation involves calculating default values based on shiitake mushroom caps phenotypic data and analyzing the correlation between these data and weight; (3) shiitake mushroom caps weight is estimated based on four highly relevant phenotypic traits, for which six mainstream machine learning algorithms were utilized to establish a weight estimation model for shiitake mushrooms. Finally, an optimal model was selected through analysis.

#### 2.3.1 KL-DexiNed algorithms

##### 2.3.1.1 Data preprocessing

The image acquisition process was influenced by variations in the positioning of shiitake mushroom caps and contamination of the background paper (e.g., water stains),resulting in inevitable background interference, as well as light and shadow disturbances. Therefore, it is imperative to preprocess visible light images of shiitake mushroom caps (Tang et al., 2023) by primarily eliminating images with significant light and shadow influence, along with substantial background interference. In this study, shiitake mushroom cap samples were uniformly positioned under consistent light source intensity to experimentally validate the robustness and accuracy of edge algorithms across different background colors (Sharma et al., 2023). Subsequently, the background exhibiting higher accuracy will be selected as the fixed reference.

##### 2.3.1.2 KL-DexiNed architecture

**Figure 5:**
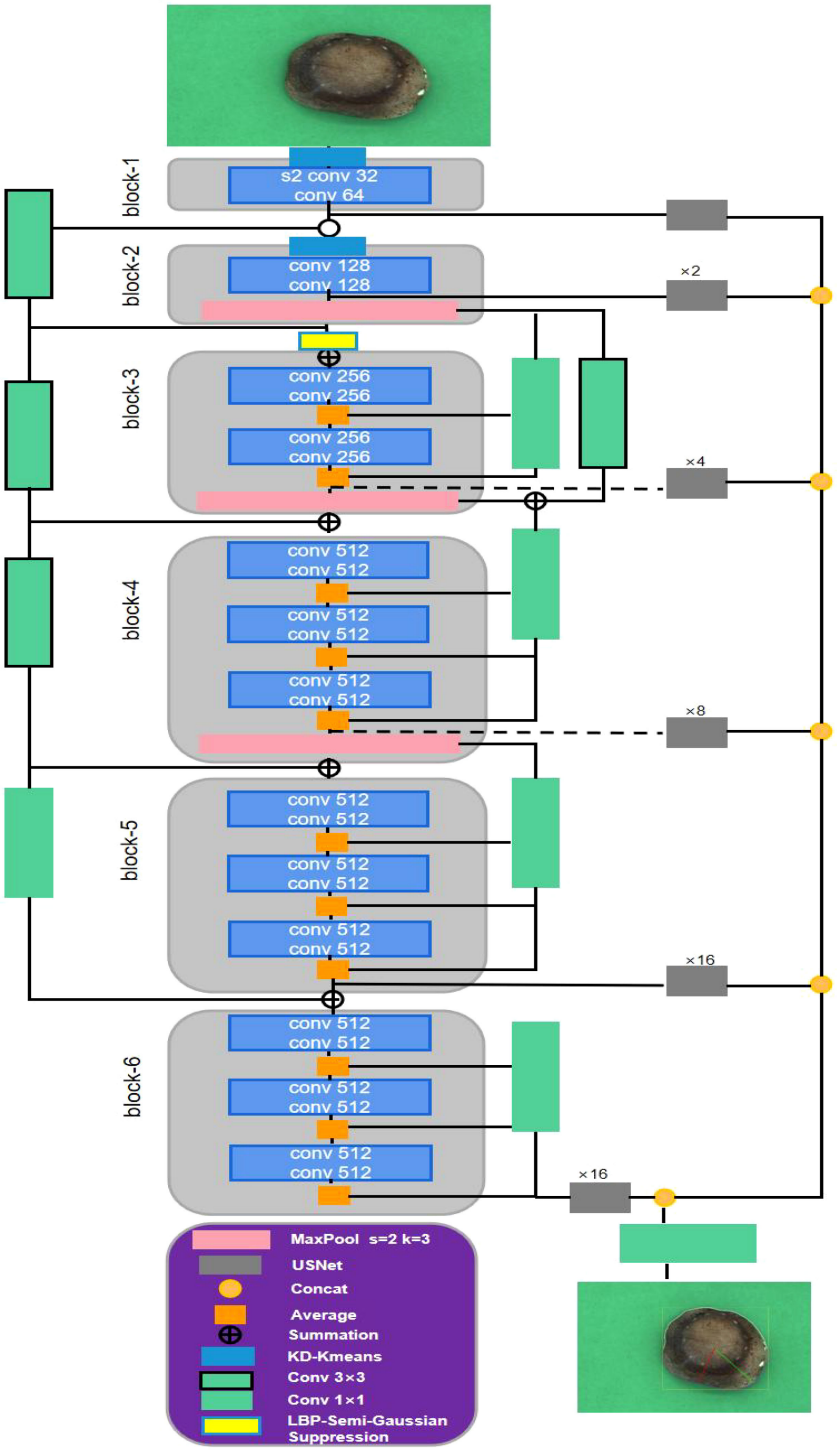
The network structure diagram of the shiitake mushroom cap edge detection based on KL-DexiNed.

Compared with traditional edge detection algorithms such as the Sobel operator and the Canny operator, DexiNed (Soria et al., 2023) can eliminate noise interference and capture the real edge contours of objects more accurately. This is because it adopts a deep neural network structure and has learned rich edge feature patterns through a large amount of training data. Meanwhile, it is capable of handling complex edge structures. Therefore, it is used as the basic network for improvement. Considering the limitations of existing methods in detecting the edges of shiitake mushroom caps, this study proposes a KL-DexiNed network that combines DexiNed and K-means algorithms. The algorithmic framework diagram is presented in **Figure 5**. **Table 2** provides a description of how the KL-DexiNed algorithm was applied for shiitake mushroom cap detection in this study.

**Table 2:** KL-DexiNed Algorithm Pseudo-Code.

Algorithm: Apply KL-DexiNed Find Cap edge
1. Read the image 2. Convert the image to grayscale 3. Apply Gaussian blur to the image to reduce noise 4. Apply the KL-DexiNed algorithm 5. Add a texture suppression module before shallow feature fusion 6. { 7. Calculate the magnitude of the gradient at each pixel 8. Threshold the gradient magnitude to obtain the final edge map 9. Initialize contour count to 0 10. Find contours in the edge map 11. For each contour found do: 12. If the contour’s area is below a predefined small area threshold: 13. Eliminate the contour 14. Else: 15. Increment the contour count 16. Return the contour count 17. }

As depicted in **Figure 5**, DexiNed enables end-to-end training without relying on weighted initialization from a pre-trained object detection model, unlike most deep learning-based edge detectors. KL-DexiNed aligns with the network backbone of DexiNed, which consists of six blocks connected by standard convolutional layers of varying sizes. During our observation while detecting shiitake mushroom cap edges, we noticed that edge features computed in shallow layers were indistinct; however, certain shiitake mushroom caps exhibited distinct pattern lines due to moisture deficiency, significantly interfering with their edge detection. To enhance the edge features of shiitake mushroom caps in shallow areas, this study employs a KD tree (K-Dimensional Tree) to optimize the K-means algorithm (Ikotun et al., 2023). The KD tree is utilized for rapid selection of initial clustering centers by choosing leaf nodes as the starting points, thereby improving both convergence speed and cluster quality of the K-means algorithm. This forms the KD-Kmeans module. The color channel is set to 2 in order to differentiate between background and shiitake mushroom caps, enabling removal of most surface patterns on the caps while understanding pixel importance and feature mapping positions. For insertion position, this study incorporates a K-means algorithm judgment before the first two blocks to extract more meaningful edge features from shiitake mushroom caps and reduce data dimensionality. Additionally, an LBP-Semi-Gaussian Suppression module is introduced prior to block-2 feature fusion which applies local binary pattern (LBP) for texture information extraction by comparing pixel gray values with their surrounding neighbors. Furthermore, Semi-Gaussian Suppression is employed to suppress texture for smaller patterns on the surface of shiitake mushroom caps, reducing computational load while maintaining accuracy. Finally, based on a minimum threshold setting, detected edge features are mapped onto the original image to obtain an accurate representation of shiitake mushroom cap edges.

##### 2.3.1.3 Size conversion factor

In this study, the test stand, shiitake mushroom cap, and standard ruler were positioned individually above the background paper. The camera lens was adjusted on the test stand to maintain a fixed distance of 270mm from the background paper. The ruler and shiitake mushroom cap were placed within the field of view area accordingly. Proportional conversion was applied to determine the scale value in relation to pixel number. To verify accuracy, scales of 5mm, 3mm, and 1mm were selected for calibration purposes. Ultimately, a conversion factor of 0.043 was derived to establish correspondence between pixel points and real sizes.

#### 2.3.2 Weight prediction models

The machine learning approach for estimating the weight of shiitake mushroom caps based on their phenotypic features effectively reduces labor (by minimizing manual weighing) and cost (by eliminating pressure sensors). This addresses the challenge of achieving a small range of error in shiitake mushroom cap weights in real-time. To achieve this, six machine learning algorithms with low computational effort and high accuracy were selected:SVR (Rante et al., 2023), LSTM (Cahuantzi et al., 2023), LSTM (Cahuantzi et al., 2023), GWO_SVM (Yan et al., 2023), Optuna-LSTM (You, 2024), WOA_BiLSTM_attention (Gao, 2023), and BiLSTM (Imani, 2023). Additionally, the input items were categorized into four groups, namely M1, M2, M3, and M4 based on their relevance. Each group was sequentially reduced by one parameter with the lowest relevance before being fed into the six machine learning algorithms.

#### 2.3.3 Evaluation methodology

For machine vision, this study compares the algorithmic edge detection results with manually drawn edges. And the algorithm is evaluated by Precision, Recall, ODS, OIS, average precision (AP), and execution rate, and F-of Precision and Recall. The specific formula is as follows:


(1) $$\text{Precision}=\frac{\text{TP}}{\text{TP}+\text{FP}}$$

(2) $$\text{Recall}=\frac{\text{TP}}{\text{TP}+\text{FN}}$$

(3) $$\text{F}=2\times \frac{\text{Precision}\times \text{Recall}}{\text{Precision}+\text{Recall}}$$

For machine learning, in order to evaluate the reliability of the model, the coefficient of determination (R²), mean absolute error (MAE),root mean square error (RMSE), and mean square error (MSE) were used in this study to evaluate the estimation results. And One subset was selected as the test set, and the remaining 4 subsets (549 samples) were combined to form the training set. The model was trained on the training set and then evaluated on the test set. This process was repeated 5 times, with each subset serving as the test set exactly once. The final performance of the model was obtained by averaging the performance metrics including R², MAE, RMSE, MSE across these 5 iterations. This approach enabled us to comprehensively assess the generalization ability of our model and obtain a more reliable evaluation of its performance.


(4) $${\text{R}}^{2}=1-\frac{\sum_{\text{i}}|({\hat{\text{y}}}_{\text{i}}-{\text{y}}_{\text{i}}{|}^{2}}{\sum_{\text{i}}|({\bar{\text{y}}}_{\text{i}}-{\text{y}}_{\text{i}}{|}^{2}}$$

(5) $$\text{RMSE}=\sqrt{\frac{1}{\text{m}}\sum_{\text{i}=1}^{\text{m}}|({\text{y}}_{\text{i}}-{\hat{\text{y}}}_{\text{i}}{|}^{2}}$$

(6) $$\text{MSE}=\frac{1}{\text{m}}\sum_{\text{i}=1}^{\text{m}}|({\text{y}}_{\text{i}}-{\hat{\text{y}}}_{\text{i}}{|}^{2}$$

(7) $$\text{MAE}=\frac{1}{\text{m}}\sum_{\text{i}=1}^{\text{m}}|({\text{y}}_{\text{i}}-{\hat{\text{y}}}_{\text{i}}|$$

Where, ${\hat{\text{y}}}_{\text{i}}$ is the predicted value, ${\text{y}}_{\text{i}}$ is the true value, ${\bar{y}}_{i}$ is the mean value. MAE can reflect the actual situation of the error of the predicted value. MSE is the expected value of the square of the difference between the model value and the observed value. RMSE is the arithmetic square root of MSE, the smaller the value the better the effect.R²is a commonly used metric for assessing the model fit goodness of fit in regression analysis.

## 3 Results

### 3.1 Edge algorithm performance

**Figure 6:**
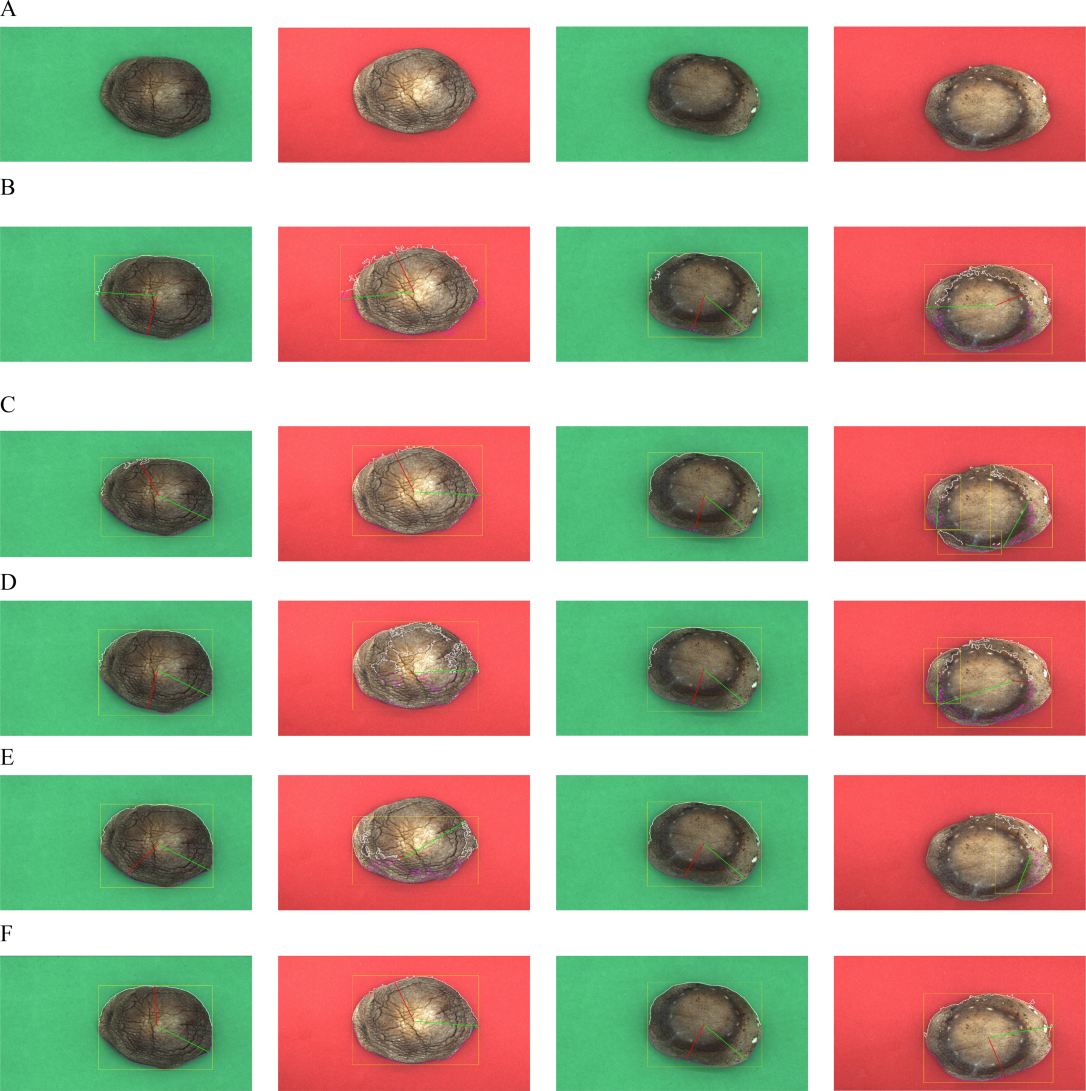
Performance of individual algorithms in different contexts. **(A)** More and less folds on the surface of shiitake mushrooms **(B)** Canny **(C)** RCF **(D)** HED **(E)** Dexined **(F)** KL-Dexined (ours).

This study presents a comparative analysis of various edge detection algorithms for accurately detecting shiitake mushroom caps against red and green backgrounds. **Figure 6** illustrates the results obtained from five different edge detection algorithms, namely Dexined, Canny (Choi and Ha, 2023), HED (Kumar, 2023), RCF (Liu et al., 2024), and KL-Dexined, when applied to images with red and green backgrounds. The analysis reveals significant variations among these algorithms in terms of edge detection accuracy, continuity of edges, and noise suppression capabilities. For the initial set of input data, shiitake mushrooms with diverse patterns and flat surfaces were selected to evaluate algorithm performance. Notably, the Dexined algorithm effectively suppresses background noise while preserving edge details using advanced image filtering and thresholding techniques; it demonstrates exceptional performance in detecting shiitake mushrooms against a green background. However, when dealing with a red background or complex patterned shiitake mushrooms specifically, the classical Canny algorithm exhibits superior noise suppression abilities while maintaining sensitivity towards edge information; it achieves high accuracy in detecting edges on green backgrounds but introduces more noise on red backgrounds where accurate edge detection becomes challenging. HED algorithm can provide more accurate edge localization when dealing with images with complex textures and high contrast edges, but it can provide more accurate edge localization in the red background. Provide more accurate edge localization, but its detection ability in low-contrast regions is insufficient. The RCF algorithm, on the other hand, is outstanding in terms of edge coherence, but may introduce some pseudo-edges in some cases, which is due to its overemphasis on the local features of the image. The KL-Dexined algorithm, which is the algorithm proposed in this study, combines the advantages of the above algorithms, and innovatively introduces a color clustering algorithm, which improves the robustness of the algorithm under different background conditions, and at the same time can reduce the interference of the surface texture of shiitake mushrooms caps on the detection algorithm. In **Figure 6F**, it can be seen that the KL-Dexined algorithm performs well in terms of both accuracy and continuity of edge detection, is not limited to changes in background color, and also achieves a better balance in terms of noise suppression, while experiments have proved that the green background has a higher robustness than the red background.

In order to further quantify the performance of each algorithm, this study employs a range of evaluation metrics such as ODS, OIS, AP, F-score, etc. Detailed quantitative statistical analysis and comparative results will be presented in subsequent sections.

**Figure 7:**
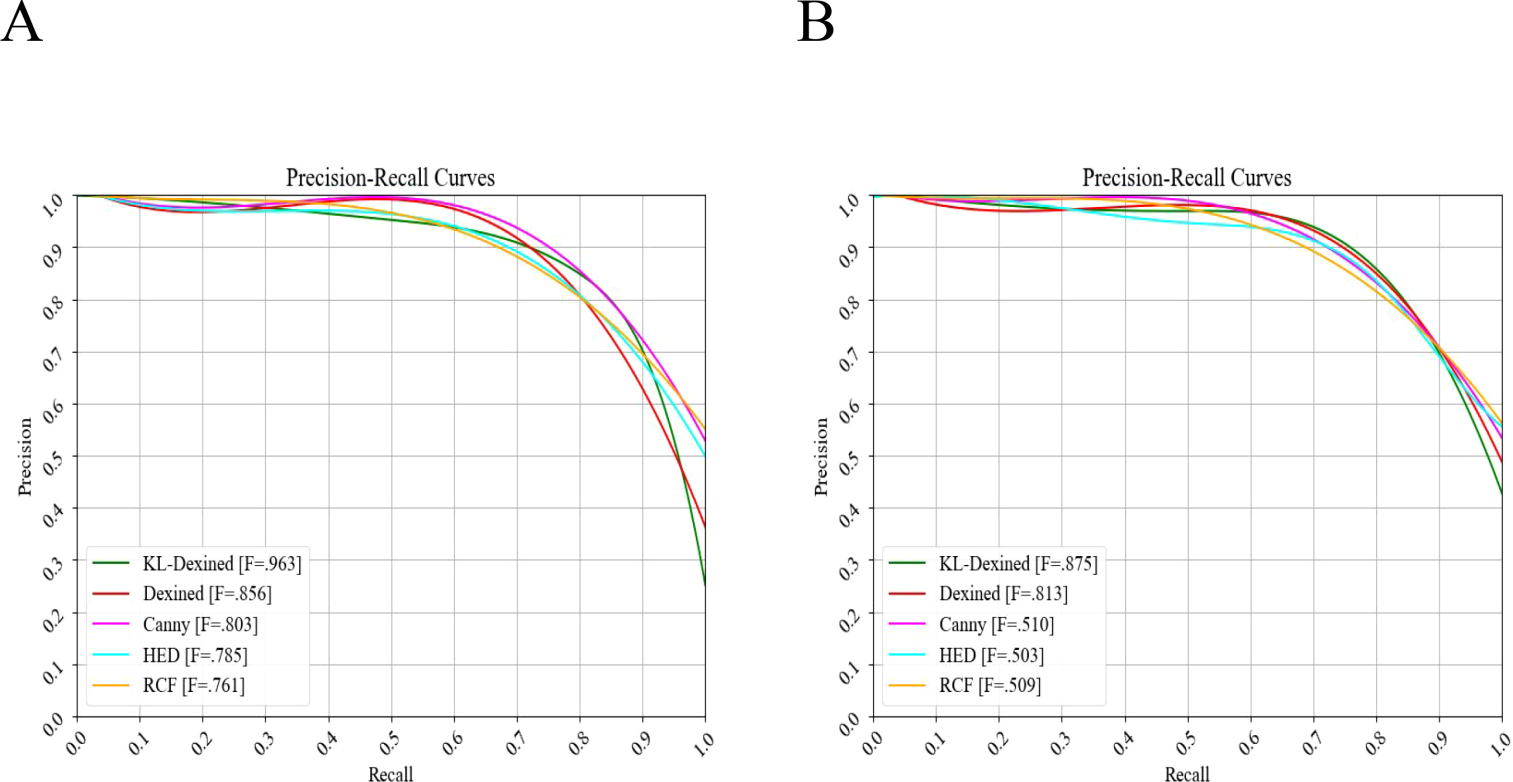
Precision-Recall curves for different algorithms. **(A)** Precision-Recall curve of Green-shiitake mushroom caps. **(B)** Precision-Recall curve of Red-shiitake mushroom caps.

**Figure 7** illustrates a performance comparison of various edge detection networks on shiitake mushroom caps with red and green backgrounds, encompassing the prominent edge detection algorithms from Canny’s algorithm publication in 1986 to present. It thoroughly evaluates their ability to detect edges on shiitake mushroom caps under different backgrounds, as presented in **Table 3**. The performance metrics include ODS, OIS, and AP. On the Green-shiitake mushroom caps dataset, the Canny algorithm proposed in 1986 achieved an ODS of 0.803 and an OIS of 0.815, with an AP reaching 0.869, demonstrating its robust detection capability. However, on the Red-shiitake mushroom caps dataset, the performance of the Canny algorithm deteriorated significantly with an ODS and OIS of only 0.510 and 0.497 respectively, along with an AP value of merely 0.533; this can be attributed to the complexity of the background for detection as well as reflective situations encountered during analysis. The HED algorithm introduced at ICCV conference in 2015 exhibited slightly inferior robustness compared to Canny on Green-shiitake mushroom caps dataset achieving an ODS and OIS values of approximately 0.785 and 0.793 respectively along with AP value arounds at about.703.On the Red-shiitake mushroom caps dataset, the HED algorithm exhibits an ODS and OIS of 0.503 and 0.516, respectively, along with an AP of 0.525, indicating its adaptability in both red and green backgrounds; however, there is still room for improvement in performance. The RCF algorithm, presented at the CVPR conference in 2017, achieves an ODS and OIS of 0.761 and 0.771,as well as an AP of 0.756 on the Green-shiitake mushroom caps dataset, demonstrating similar but slightly reduced performance compared to HED on this specific dataset. On the Red-shiitake mushroom caps dataset, RCF maintains stability with an ODS and OIS of 0.509 and 0.518 respectively, accompanied by an AP of 0.523. The Dexined algorithm significantly improves performance on the Green-shiitake mushroom caps dataset with impressive values for ODS (0.856), OIS (0.875), and AP (0.885),highlighting its superiority in edge detection tasks. The Dexined algorithm also performs well on the Red-shiitake mushroom caps dataset with notable scores for ODS (0.813), OIS (0.836) and AP (0.855), further showcasing its ability to generalize across different contexts.

**Table 3:** Performance comparison of different edge detection networks.

Models	Pub.’Year	Train on	ODS	OIS	AP
Canny	PAMI’86	Green-shiitake mushroom caps	.803	.815	.869
HED	ICCV’15	Green-shiitake mushroom caps	.785	.793	.703
RCF	CVPR’17	Green-shiitake mushroom caps	.761	.771	.756
Dexined	WACV’20	Green-shiitake mushroom caps	.856	.875	.885
KL-Dexined (ours)	—	Green-shiitake mushroom caps	.963	.935	.971
Canny	PAMI’86	Red-shiitake mushroom caps	.510	.497	.533
HED	ICCV’15	Red-shiitake mushroom caps	.503	.516	.525
RCF	CVPR’17	Red-shiitake mushroom caps	.509	.518	.523
Dexined	WACV’20	Red-shiitake mushroom caps	.813	.836	.855
KL-Dexined (ours)	—	Red-shiitake mushroom caps	.875	.869	.881

Finally, KL-Dexined achieved the highest ODS and OIS scores of 0.963 and 0.935, respectively, on the Green-shiitake mushroom caps dataset, along with an AP of 0.971, demonstrating superior performance in edge detection tasks. Moreover, our algorithm exhibited commendable results on the Red-shiitake mushroom caps dataset with ODS and OIS scores of 0.875 and 0.869,respectively, as well as an AP score of 0.881; these findings further validate the effectiveness and robustness of our proposed algorithm while also highlighting its suitability for shiitake mushroom cap detection when a green background is employed.

### 3.2 Predicted results for key phenotypic traits

**Figure 8:**
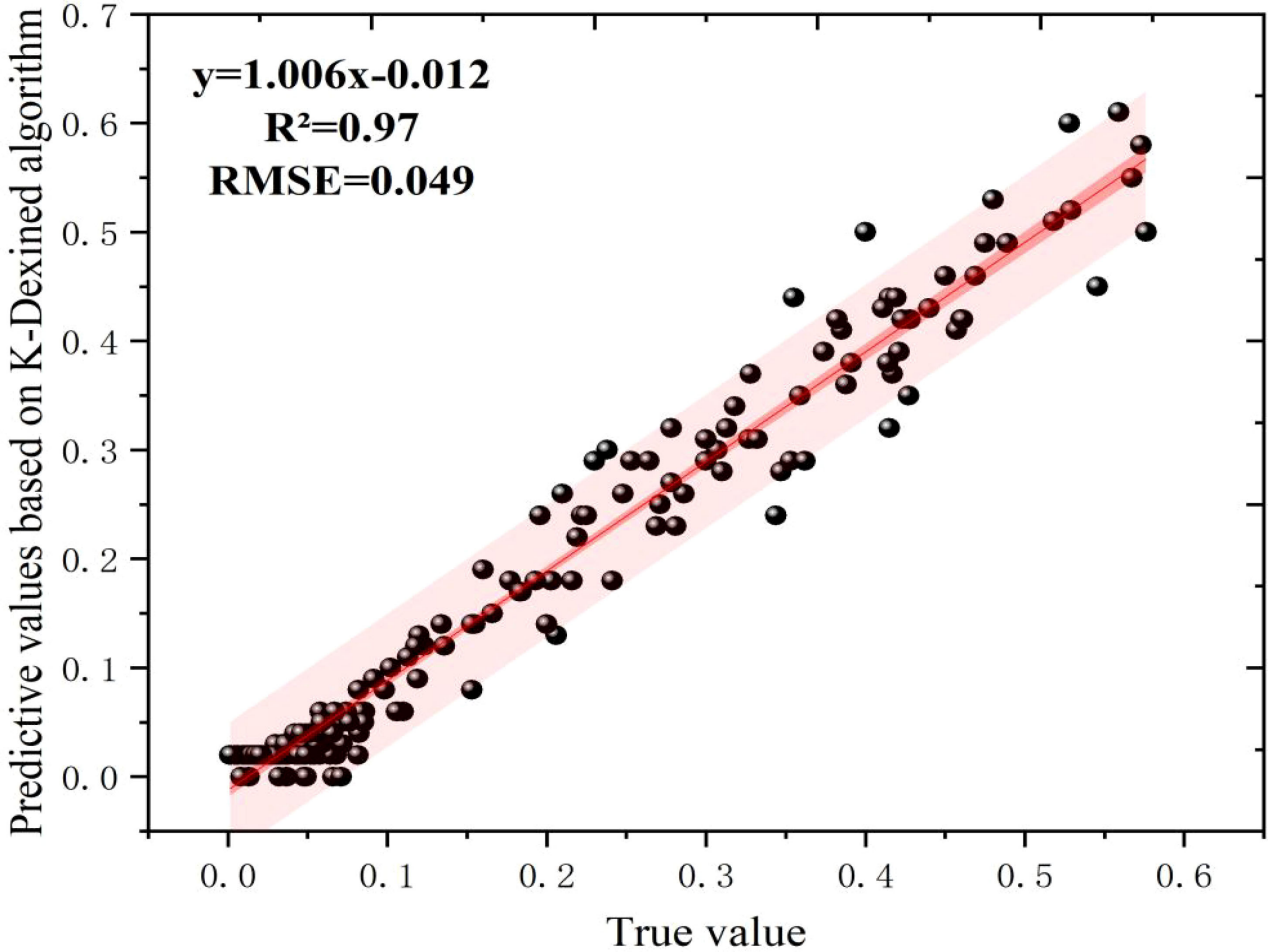
Comparison of predicted and true values based on KL-Dexined edge feature detection algorithm.

According to the rules in **Table 1**, we calculated the key phenotypic real values of 686 shiitake mushrooms caps samples, and the data are shown in **Table 4**. Meanwhile, according to the results of KL-Dexined edge feature detection, in this study, we used OpenCV to draw the external rectangular box, center of mass, long and short axes, etc., which were inputted into the Green-shiitake mushroom caps dataset, and the results of 11 key phenotypic traits were obtained. The predicted values of the algorithm were compared with the real measurements after normalization, as shown in **Figure 8**, and the R²between the predicted values and the real values was obtained as 0.97, and the RMSE was 0.049, which proved experimentally that the algorithm of this study has the ability to predict the key phenotypic traits.

**Table 4:** True values of phenotypic characteristics of shiitake mushrooms caps.

Phenotype category	Average	Maximum	Minimum	Standard deviation	Standard Score Maximum	Standard Minimum
External rectangular length	60.1	69.24	52.86	4.65	1.98	-1.55
External rectangular width	60.02	66.86	53.57	3.89	1.76	-1.66
Roundness	0.81	0.9	0.57	0.069	1.34	-3.38
Area	208.32	244.58	182.93	16.54	2.19	-1.54
Perimeter	2782.4	3351.2	2215.15	343.02	1.66	-1.65
Long axis	39.75	43.15	32.17	3.51	0.97	-2.16
Short axis	34.38	40.38	24.83	4.2	1.43	-2.27
Red mean	103.42	161.97	51.16	27.48	2.13	-1.90
Green mean	68.64	113.6	30.161	22.40	2.01	-1.72
Blue mean	41.91	74.33	19.13	15.37	2.11	-1.48
Greyscale mean	70.99	114.18	33.15	21.52	2.01	-1.76

At the same time, we quantitatively analyzed the measured phenotypic features to explore the differences between different phenotypic categories. For the external rectangular length, the average is 60.1, the maximum is 69.24, the minimum is 52.86, and the standard deviation is 4.65, with standard score ranging from a maximum of 1.98 to a minimum of - 1.55. For the external rectangular width, the average is 60.02, the maximum is 66.86, the minimum is 53.57, the standard deviation is 3.89, and the standard score ranges from 1.76 to - 1.66. The roundness has an average of 0.81, a maximum of 0.9, a minimum of 0.57, a standard deviation of 0.069, and its standard score ranges from 1.34 to - 3.38. The area has an average of 208.32, a maximum of 244.58, a minimum of 182.93, a standard deviation of 16.54, and the standard score ranges from 2.19 to - 1.54. The perimeter has an average of 2782.4, a maximum of 3351.2, a minimum of 2215.15, a standard deviation of 343.02, and the standard score ranges from 1.66 to - 1.65. The long axis has an average of 39.75, a maximum of 43.15, a minimum of 32.17, a standard deviation of 3.51, and the standard score ranges from 0.97 to - 2.16. The short axis has an average of 34.38, a maximum of 40.38, a minimum of 24.83, a standard deviation of 4.2, and the standard score ranges from 1.43 to - 2.27. The red mean has an average of 103.42, a maximum of 161.97, a minimum of 51.16, a standard deviation of 27.48, and the standard score ranges from 2.13 to - 1.90. The green mean has an average of 68.64, a maximum of 113.6, a minimum of 30.16, a standard deviation of 22.40, and the standard score ranges from 2.01 to - 1.72. The blue mean has an average of 41.91, a maximum of 74.33, a minimum of 19.13, a standard deviation of 15.37, and the standard score ranges from 2.11 to - 1.48. The greyscale mean has an average of 70.99, a maximum of 114.18, a minimum of 33.15, a standard deviation of 21.52, and the standard score ranges from 2.01 to - 1.76.

In summary, the data in the table provide key traits of shiitake mushrooms cap phenotypes, including color information and scale information. These statistical information are important for subsequent estimation of shiitake mushroom caps weight.

### 3.3 Correlation between weight and key phenotypes

**Figure 9:**
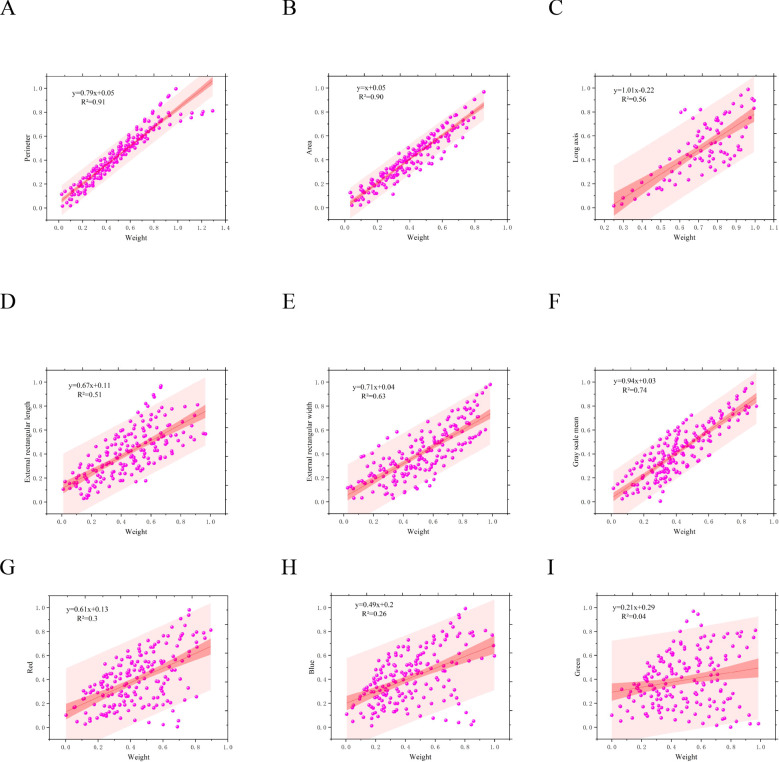
Analysis of the correlation between phenotypic indicators and weight of shiitake mushroom caps. **(A)** Perimeter **(B)** Area **(C)** Long axis **(D)** External rectangular length **(E) ** External rectangular width **(F)** Greyscale mean **(G)** Red mean **(H)** Green mean **(I)** Blue mean.

In this study, to establish the relationship between phenotypic information and weight of shiitake mushrooms, we examined 11 phenotypic indicators of shiitake mushroom caps in correlation with weight (**Figure 9**). The obtained correlations ranked from highest to lowest as follows: Perimeter (0.91), Area (0.9), Gray scale mean (0.74), External rectangular width (0.63), Long axis (0.56),External rectangular length (0.51), Red channel intensity (0.3), Blue channel intensity (0.26), Green channel intensity (0.04). Two remaining indices exhibited correlations lower than 0.01 and were therefore excluded from consideration. Additionally, four parameter combinations were designed based on these correlations, as shown in **Table 5**, and inputted into various machine learning algorithms including WOA_BiLSTM_attention, SVR, BiLSTM, LSTM, GWO_SVM, and Optuna-LSTM for predicting the weight of shiitake mushroom caps.

**Table 5:** 4 different input combinations.

Combinatorial	M1	M2	M3	M4
Input item	Area、Perineter、External rectangular width、Long axis	Area、Perineter、External rectangular width	Area、Perineter	Perineter

### 3.4 Weight prediction model performance

**Figure 10:**
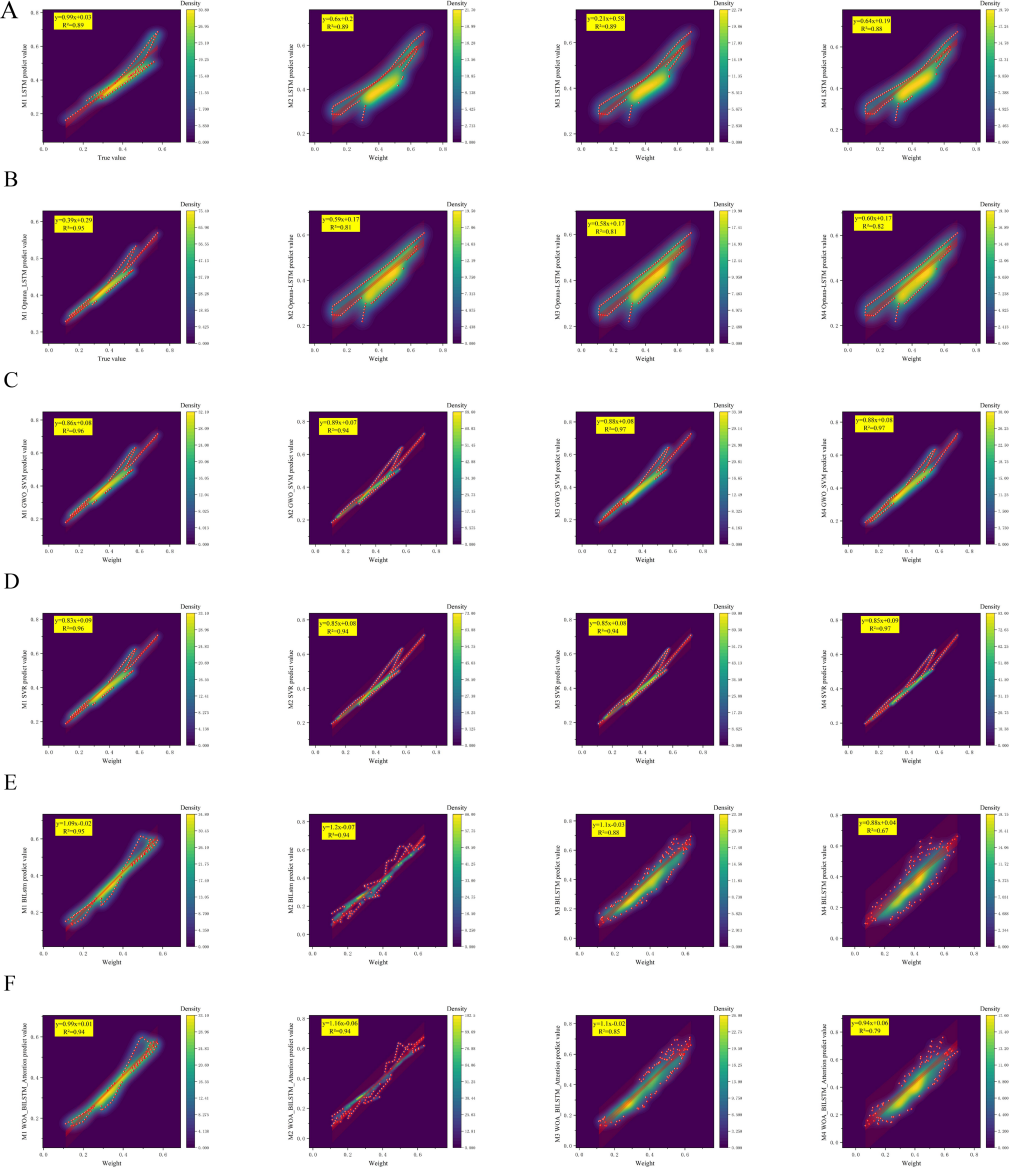
Estimated weight of shiitake mushrooms caps with different phenotypic characterization inputs. **(A)** The performance of the LSTM algorithm when the input items are from the M1-M4 groups. **(B)** The performance of the Optuna_LSTM algorithm when the input items are from the M1-M4 groups. **(C)** The performance of the GWO_SVM algorithm when the input items are from the M1-M4 groups. **(D)** The performance of the SVR algorithm when the input items are from the M1-M4 groups. **(E)** The performance of the BILSTM algorithm when the input items are from the M1-M4 groups. **(F)** The performance of the WOA_BILSTM_attention algorithm when the input items are from the M1-M4 groups.

As can be seen from **Figure 10**, the R²of GWO_SVM model reaches the highest of 0.97 in the input M3 and M4 groups, and the R²of GWO_SVM in the M1 group with only one input term with the true value also reaches 0.96, which is obviously better than other machine learning algorithms. In terms of error, as shown in **Table 6**, it demonstrates the performance comparison of the six machine learning models under different input combinations in the three evaluation metrics of MSE, mean absolute error MAE and RMSE. With the reduction of input variables, the RMSE of each algorithm increases, while the MAE and MSE do not change significantly. The models selected for this study include LSTM, SVR, BiLSTM, WOA_BiLSTM_attention,Optuna_LSTM, and GWO_SVM. Under the M1 and M2 input combinations, all the models exhibit low error values, with SVR and GWO_SVM maintaining the lowest error levels in all the evaluative metrics, especially The performance of GWO_SVM on MAE and RMSE is especially outstanding, with 0.033 and 0.040, respectively. However, when the input combination is changed to M3,the performance of the BiLSTM model on RMSE slightly decreases to 0.056, while the WOA_BiLSTM_attention model maintains a lower error level. With the M4 input combination, the BiLSTM model has the highest error values with MSE, MAE and RMSE of 0.007, 0.063 and 0.080, respectively, while the GWO SVM again exhibits the lowest error values of 0.002, 0.032 and 0.039, respectively.

**Table 6:** Performance comparison of 6 machine learning models with 4 input combinations.

Combinatorial	Algorithm	MSE	MAE	RMSE
M1	LSTM	0.002	0.036	0.042
	SVR	0.002	0.035	0.042
	BiLSTM	0.003	0.025	0.040
	WOA_BiLSTM_attention	0.002	0.027	0.041
	Optuna_LSTM	0.003	0.042	0.051
	GWO_SVM	0.002	0.033	0.040
M2	LSTM	0.006	0.059	0.077
	SVR	0.002	0.034	0.041
	BiLSTM	0.002	0.030	0.041
	WOA_BiLSTM_attention	0.002	0.026	0.039
	Optuna_LSTM	0.006	0.062	0.074
	GWO_SVM	0.002	0.033	0.041
M3	LSTM	0.006	0.060	0.079
	SVR	0.002	0.033	0.041
	BiLSTM	0.003	0.043	0.056
	WOA_BiLSTM_attention	0.003	0.041	0.053
	Optuna_LSTM	0.006	0.062	0.075
	GWO_SVM	0.002	0.033	0.038
M4	LSTM	0.006	0.058	0.076
	SVR	0.002	0.033	0.040
	BiLSTM	0.007	0.063	0.080
	WOA_BiLSTM_attention	0.005	0.058	0.073
	Optuna_LSTM	0.005	0.062	0.074
	GWO_SVM	0.002	0.032	0.039

**Figure 11:**
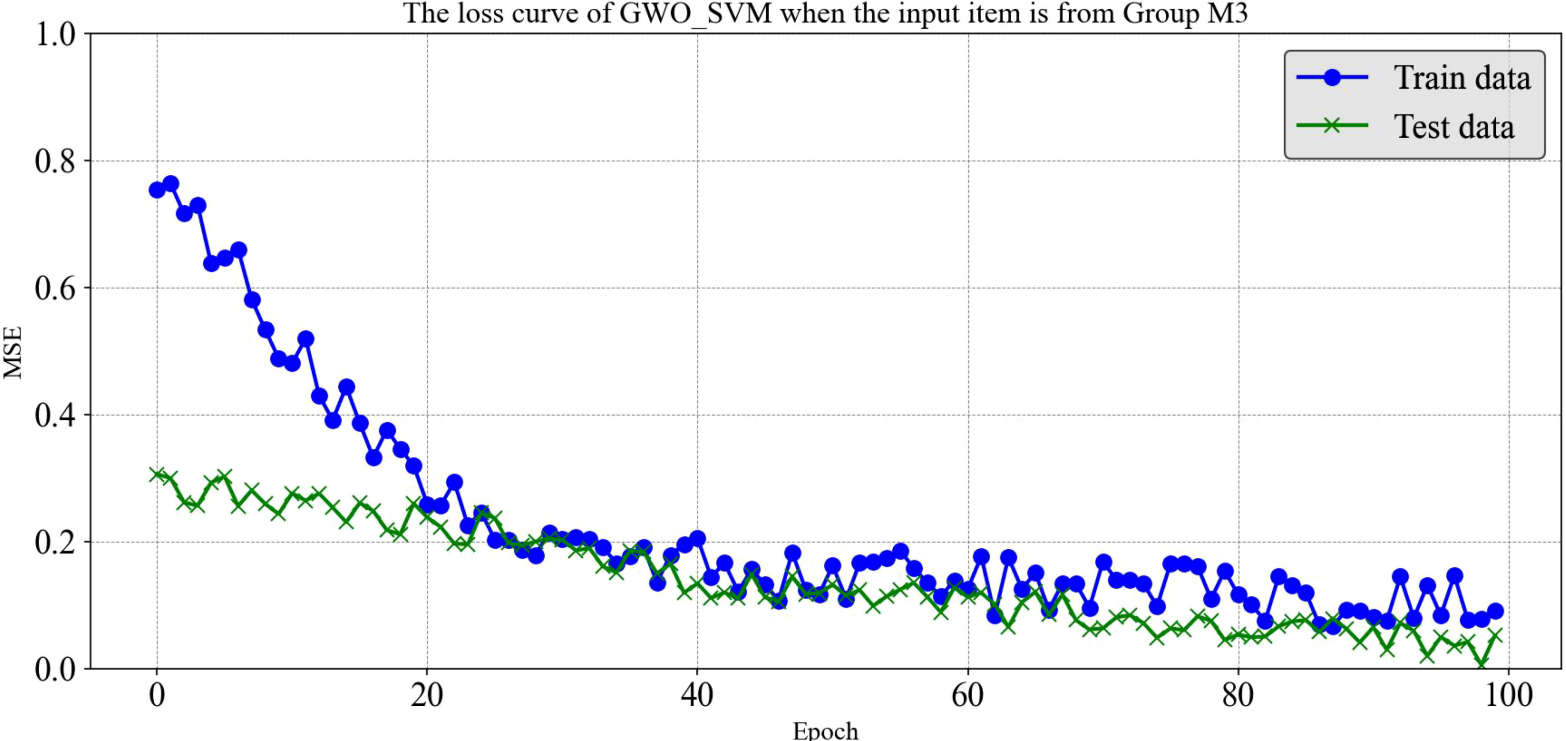
The loss curve of GWO_SVM when the input item is from Group M3.

It is worth noting that although LSTM and Optuna_LSTM exhibit higher error values in certain cases, they demonstrate reduced variability under diverse input combinations, indicating a degree of robustness. Furthermore, the GWO_SVM model consistently maintains a low error level across all input combinations, highlighting its exceptional adaptability and generalization to the input data. Notably, the GWO_SVM algorithm outperforms others when subjected to the M3 set of parameter inputs, achieving an impressive R² value of 0.97 between predicted and true values with a minimal error rate of only 0.038.It can be seen from **Figure 11** that the loss situations of the training set and the testing set in the loss curve of GWO_SVM when the input item is from Group M3.

## 4 Discussion

In this study, a novel edge detection algorithm, KL-Dexined, was successfully developed, and considering that the accuracy of the algorithm may change under different backgrounds (Choi and Ha, 2023), this study verified its accuracy under different background conditions through a series of experiments. The algorithm in this study demonstrated high accuracy and continuity in the edge detection task of shiitake mushrooms caps on both red and green backgrounds, while achieving a good balance of noise suppression. Thanks to the introduction of the innovative color clustering technique in the algorithm, it not only improves the robustness of the algorithm under different background conditions, but also reduces the interference of the surface texture of shiitake mushrooms caps on the detection algorithm. In addition, this study explored the key phenotypic traits of shiitake mushrooms caps, including the length and width of the outer tangent rectangle, roundness, area, perimeter, long and short axes, etc., and predicted these traits using the edge features detected by the KL-Dexined algorithm. By analyzing the results in comparison with the ground-truth measurements, this study found that the correlation between the algorithm’s predicted values and the real values was very high, which indicated that the conjecture of this study (a positive correlation between the weight of the cap of shiitake mushrooms and the phenotype) was correct.

In terms of weight prediction, the highest correlation between perimeter, area, outer rectangle width and long axis of shiitake mushrooms caps and weight was found considering weight and key phenotypic traits. Based on these findings, four different parameter combinations were designed and machine learning models were used to validate the possibility of predicting the weight of shiitake mushrooms caps. The experimental results show that the GWO_SVM model performs best with M3 and M4 sets of parameter inputs, with an R²value of 0.97 between the predicted value and the true value, and a root mean square error (RMSE) of only 0.038, whereas, especially under the M4 group input, the LSTM family of algorithms had a relatively high error, with LSTM, BiLSTM, WOA_BiLSTM_attention, and Optuna_LSTM all having RMSE above 0.073, the reason for not having SVR,GWO_SVM algorithms perform well, which may be related to the size of the dataset in this study, and deep learning based machine learning methods do not take advantage of their advantages under small samples (Ammi et al., 2023).

Despite the remarkable results of this study, there are still some limitations. For example, the adaptability of the algorithm to different background conditions has not been fully validated. In addition, this study mainly focused on the phenotypic characterization of shiitake mushrooms caps, and the characterization of stems and segments has not been carried out. Future studies will be extended to the shiitake mushrooms’ stems as well as slices to further improve the generalization ability of the algorithm.

## 5 Conclusion

In this study, a machine vision-based and shiitake mushrooms cap weight prediction method is proposed, in which deep learning techniques, traditional OpenCV algorithms, and machine learning algorithms are used. In terms of scale detection, this study proposes the KL-DexiNed algorithm and compares it with other mainstream edge detection algorithms, and finally obtains the optimal threshold of the detected image (OIS) of 93.5%, the fixed contour threshold (ODS) of 96.3%, and the average precision (AP) of 97.1%, and maps it to the original image to obtain the edge map of shiitake mushrooms caps, and calculates the corresponding key based on the edge phenotypic metrics. In terms of phenotypic metrics to predict the weight, this study found the four phenotypic metrics with the highest correlation, which are area, perimeter, area, external rectangle width, and long axis. And divided into 4 groups to verify the effect of defaultization calculation on predicting the weight of shiitake mushrooms cap. Finally, we found that the GWO_SVM algorithm performs the best under the parameter inputs of M3 group, and its R² between predicted and true values is 0.97, while the RMSE is 0.038. It proves that our proposed phenotypic metrics estimation of shiitake mushrooms cap method is effective. Notably, the proposed method also represents a significant breakthrough in terms of practical application. It presents a machine vision-based rapid acquisition method for key traits of shiitake mushrooms caps, which can effectively reduce the workload of breeding personnel. Traditional manual measurement of shiitake mushrooms cap traits is time-consuming, labor-intensive and error-prone. By contrast, this method utilizes an automated image acquisition and intelligent analysis system, enabling rapid processing of multiple samples, accurate acquisition of multi-dimensional trait information and generation of reports. It substantially shortens the acquisition cycle, reduces human errors, allows breeding personnel to concentrate on core work, improves breeding efficiency and quality, and promotes the modernization of the shiitake mushrooms breeding industry. In the future, we will conduct research on the phenotypic indexes for shiitake mushrooms stems and slices.

## Data Availability

The raw data supporting the conclusions of this article will be made available by the authors, without undue reservation.
